# A digital volumetric tomography (DVT) study in the mandibular molar region for miniscrew placement during mixed dentition

**DOI:** 10.1590/2176-9451.20.2.055-060.oar

**Published:** 2015

**Authors:** Mayur S. Bhattad, Sudhindra Baliga, Pavan Vibhute

**Affiliations:** 1 Senior lecturer, Sharad Pawar Dental College and Hospital, Department of Pedodontics and Preventive Dentistry, Sawangi, Wardha, Maharashtra, India; 2 Professor, Sharad Pawar Dental College and Hospital, Department of Pedodontics and Preventive Dentistry, Sawangi, Wardha, Maharashtra, India; 3 Associate professor, Sharad Pawar Dental College and Hospital, Department of Orthodontics, Sawangi, Wardha, Maharashtra, India

**Keywords:** Miniscrews, Digital volumetric tomograph, Inter-radicular bone, Cortical bone

## Abstract

**OBJECTIVE:**

To assess bone thickness for miniscrew placement in the mandible during mixed dentition by using digital volumetric tomograph (DVT).

**MATERIAL AND METHODS:**

A total of 15 healthy patients aged 8-10 years old, with early exfoliated mandibular second deciduous molar, were included. DVT images of one quadrant of the mandible were obtained using Kodak extraoral imaging systems and analyzed by Kodak dental imaging software. The error of the method (EM) was calculated using Dahlberg's formula. Mean and standard deviation were calculated at 6 and 8 mm from the cementoenamel junction (CEJ).Paired t-test was used to analyze the measurements.

**RESULTS:**

Buccal cortical bone thickness, mesiodistal width and buccolingual bone depth at 6 mm were found to be 1.73 + 0.41, 2.15 + 0.49 and 13.18 + 1.22 mm, respectively; while at 8 mm measurements were 2.42 + 0.34, 2.48 + 0.33 and 13.65 + 1.25 mm, respectively. EM for buccal cortical bone thickness, mesiodistal width and buccolingual bone depth was 0.58, 0.40 and 0.48, respectively. The difference in measurement at 6 and 8 mm for buccal cortical plate thickness (P < 0.05) and buccolingual bone thickness (P < 0.05) was found to be significant, whereas for mesiodistal width it was insignificant (P > 0.05).

**CONCLUSION:**

Bone thickness measurement has shown promising evidence for safe placement of miniscrews in the mandible during mixed dentition. The use of miniscrew is the best alternative, even in younger patients.

## Abstract (pt)

**OBJETIVO:**

avaliar, por meio de tomografia volumétrica digital (TVD), a espessura óssea necessária para a instalação de mini-implante na arcada inferior durante a fase de dentição mista.

**MÉTODOS:**

um total de 15 pacientes saudáveis, com idades entre 8 e 10 anos, com segundo molar inferior decíduo irrompido recentemente, foram incluídos no presente estudo. Imagens de TVD da hemiarcada inferior foram obtidas utilizando sistemas de imagens extrabucais Kodak. As imagens foram analisadas por meio do programa de imagens Kodak. O erro do método (EM) foi calculado utilizando a fórmula de Dahlberg. Médias e desvios-padrão foram calculados de 6 a 8mm aquém da junção amelocementária. O teste *t* foi utilizado para a análise das medidas.

**RESULTADOS:**

a espessura do osso cortical vestibular, largura mesiodistal e profundidade óssea vestibulolingual, a 6mm, foram de 1,73 + 0,41; 2,15 + 0,49; e 13,18 + 1,22 mm, respectivamente. Já a 8mm, os valores foram de 2,42 + 0,34; 2,48 + 0,33; e 13,65 + 1,25mm. O EM para a espessura do osso cortical vestibular, largura mesiodistal e profundidade óssea vestibulolingual foi de 0,58, 0,40 e 0,48mm, respectivamente. A diferença entre as medidas a 6 e 8mm para a espessura do osso cortical vestibular (p < 0,05) e a espessura óssea vestibulolingual (p < 0,05) foi significativa, embora não tenha sido significativa para a largura mesiodistal (p < 0,05).

**CONCLUSÃO:**

a mensuração da espessura óssea demonstra evidências promissoras para a segura instalação de mini-implantes na arcada inferior e na fase de dentição mista. O uso de mini-implantes tem se mostrado a melhor alternativa, mesmo nos casos de pacientes mais jovens.

## INTRODUCTION

Maintenance of arch length during the primary, mixed and permanent dentition is of great significance for the normal development of future occlusion because premature loss of primary teeth due to caries, trauma, ectopic eruption, or other causes may lead to undesirable tooth movements of primary and/or permanent teeth including loss of arch length.^1^ Space management is a key responsibility of dental practitioners who are concerned about monitoring the developing dentition, as the loss of arch length may lead to problems, such as crowding, dental impaction, crossbite formation, and dental midline discrepancies.^2^ The use of space maintainers/retainers are advocated to maintain or regain lost arch length and may potentially obviate the need for later extractions and/or complex orthodontic treatment, hence space management continues to play a vital role in Dentistry.^3^ However, these space maintaining devices in routine practice have shown appreciable adverse effects, such as plaque accumulation, dental caries, dislodged or broken appliances, interference with successor eruption, undesirable tooth movement and soft tissue impingement.^2^^,^^45^

In recent years, a new treatment method using miniscrews has been developed and applied to clinical orthodontic treatment. This technique enabled tooth movement that was impossible with conventional orthodontic treatment and served as an alternative method for absolute orthodontic anchorage.^6^^,^ 7 Thus, miniscrews may have the potential to aid comprehensive space management and to overcome the disadvantages of conventional space maintaining devices.

Miniscrews offer the advantages of lower cost, smaller size, easy surgical placement/removal procedure, no additional laboratory work and minimum waiting period for osseointegration.^7^^,^ 8 Numerous anatomic sites for miniscrew placement have been proven in adults; however, very few data are available for the mixed dentition age group.^6^ The scope of miniscrews in Pediatric Dentistry for space maintenance and as an anchorage device in the late mixed dentition period may be possible and needs to be evaluated. Hence, this study aimed to assess the mesiodistal bone width, buccal cortical plate thickness and buccolingual bone thickness in the posterior region of the mandible for placement of miniscrews during mixed dentition.

## MATERIAL AND METHODS

The study protocol was approved by DMIMS, Sawangi, Wardha, Mahrashtra state, India Institutional Review Board and an informed consent form was signed by parents/guardians accompanying the patients prior to the digital volumetric tomographic (DVT) scan. A total of 15 healthy patients, aged 8-10 years old, with early or recently exfoliated mandibular second deciduous molar and 2-4 mm bone covering erupting mandibular second premolar were included in the study. Patients with severe facial or dental asymmetries, systemic diseases or bone abnormalities, significant medical or dental history, vertical or horizontal periodontal bone loss were excluded.^6^^,^ 9 ^,^ 10

Digital volumetric tomographic images of one quadrant of the mandible in all 15 patients were obtained using Kodak 9000 extraoral imaging system. Either the right or left quadrant of the mandible was randomly chosen for measurement taking, as it was previously proven that there were no differences in cortical bone thickness between sides of the jaws.^11^^,^ 12

## DATA ANALYSIS

**Figure 1 -:**
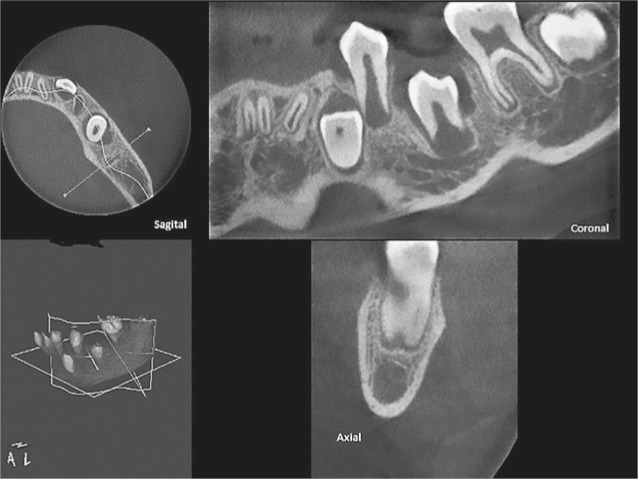
Sagittal, coronal and axial slices.

**Figure 2 -:**
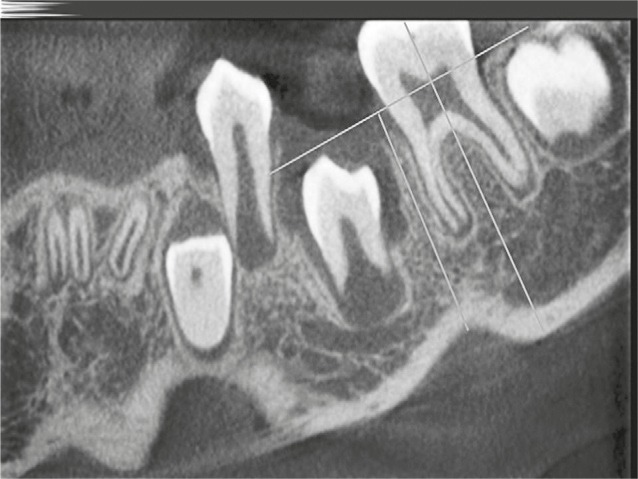
Vertical and horizontal reference plane.

**Figure 3 -:**
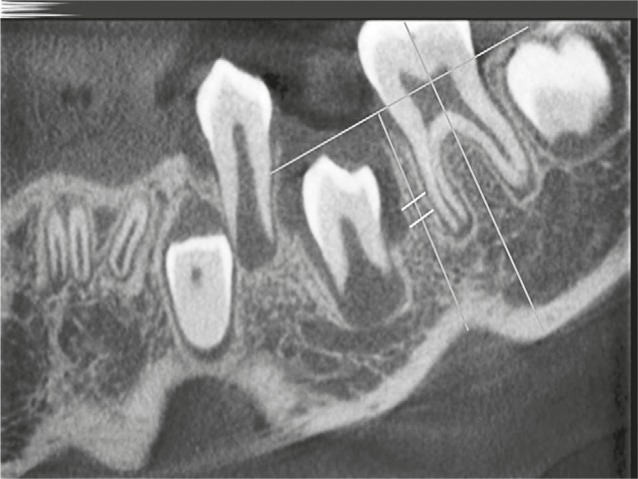
Mesiodistal bone width at 6 and 8 mm.

**Figure 4 -:**
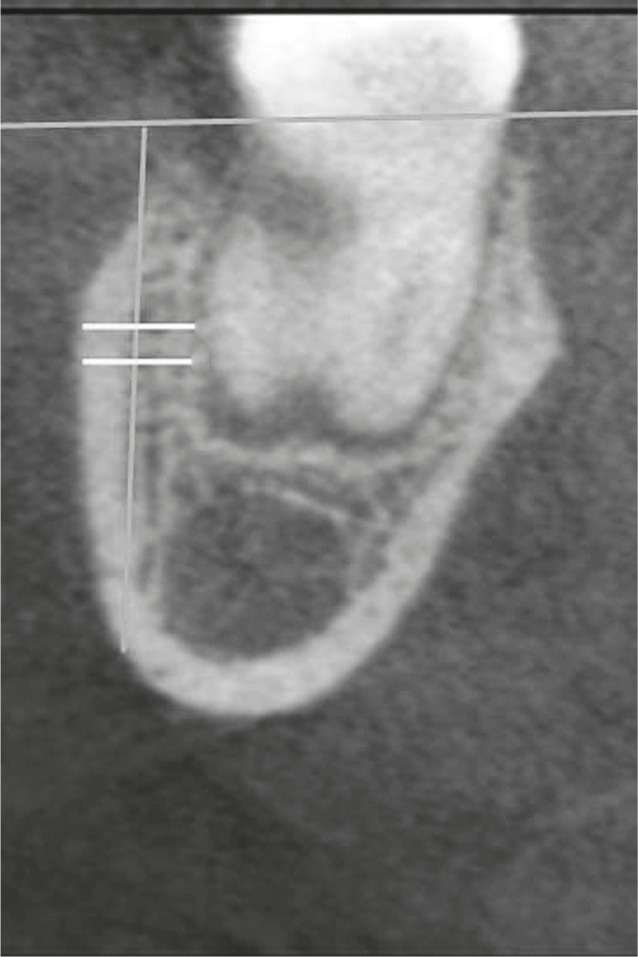
Buccal cortical bone thickness at 6 mm and 8 mm.

**Figure 5 -:**
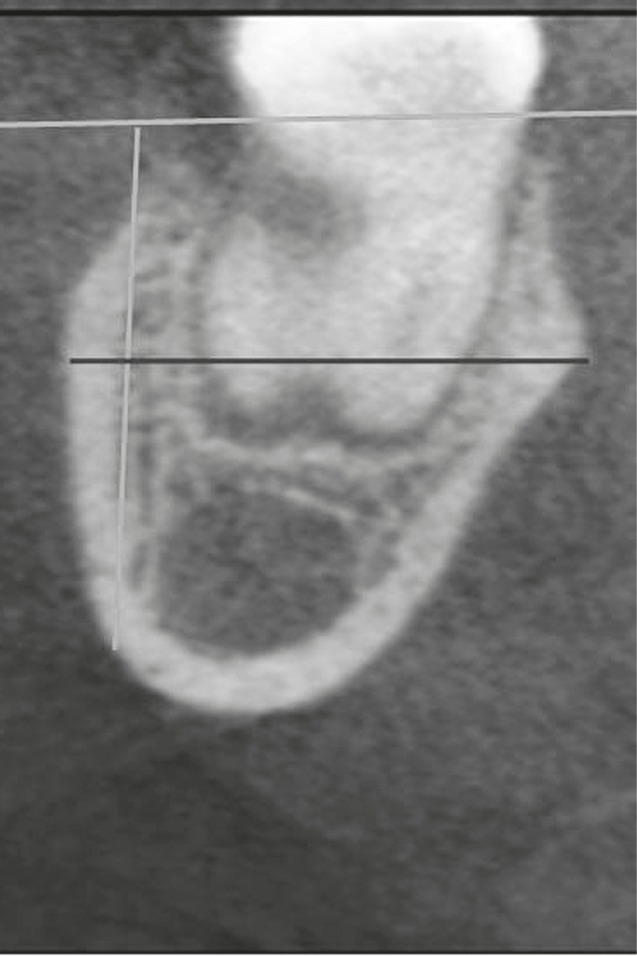
Buccolingual bone depth at 6 mm and 8 mm.

The images obtained were analyzed by Kodak dental imaging software (3D module V 2.2). At the time of measurements, scanned images were oriented in all three planes: sagittal, axial and coronal. In the posterior inter-radicular areas of the mandible, the sagittal slice was used to locate the inter-radicular area of interest for measurements (Fig 1). The vertical reference plane was made parallel to the long axes of the roots, and the horizontal reference plane was marked along the cementoenamel junction (CEJ) of permanent mandibular first molar^10^ (Fig 2). Measurements were carried out at 6 and 8 mm apical to the cementoenamel junction. Mesiodistal bone width in the mandibular first molar region was measured in sagittal slice (Fig 3) whereas the thickness of the buccal cortical plate (Fig 4) and buccolingual bone thickness or depth was measured in the areas between the second premolar and first molar in the coronal slice (Fig 5).

## STATISTICAL ANALYSIS

Data obtained for measurements at 6 mm and 8 mm were statistically analyzed by means of paired t-test. The scanned images were measured by the same observer after a two week interval. The error of the method (EM) calculations were carried out by means of Dahlberg's formula.^6^

## RESULTS

Of the 15 images obtained, three were discarded due to poor image quality. Mean and standard deviation for each of the variables were calculated. Mesiodistal bone width measurements at 6 mm and 8 mm ranged from 1.3 to 2.9 mm. Results for buccal cortical plate thickness and buccolingual bone depth ranged between 1.5 - 2.9 mm and 11.9 - 15.4 mm, respectively. Mean values for mesiodistal bone width, buccal cortical plate thickness and buccolingual bone depth at 8 mm were found to be sufficient for miniscrews placement with a diameter of 1.2 - 1.4 mm and length of 10 - 14 mm (Table 1).

**Table 1 -:** Mean and standard deviation for mesiodistal bone width, buccal cortical plate thickness and buccolingual bone depth measurements.

Patient	Mesiodistal width		Buccal cortical plate		Buccolingual bone thickness	
	6 mm	8 mm	6 mm	8 mm	6 mm	8 mm
1	2.3 mm	2.6 mm	2.0 mm	2.9 mm	13.1 mm	13.7 mm
2	2.7 mm	2.6 mm	1.5 mm	2.6 mm	11.9 mm	12.1 mm
3	1.3 mm	2.2 mm	1.9 mm	1.9 mm	15.4 mm	15.4 mm
4	2.3 mm	2.8 mm	1.8 mm	2.4 mm	12.4 mm	12.8 mm
5	2.4 mm	2.9 mm	2.1 mm	2.5 mm	13.5 mm	14.8 mm
6	1.9 mm	2.6 mm	1.9 mm	2.2 mm	12.8 mm	13.1 mm
7	2.0 mm	2.8 mm	1.8 mm	2.6 mm	13.4 mm	15.1 mm
8	2.1 mm	2.7 mm	2.2 mm	2.7 mm	13.6 mm	14.3 mm
9	1.9 mm	2.4 mm	1.9 mm	2.5 mm	13.4 mm	13.7 mm
10	2.4 mm	2.8 mm	1.8 mm	2.3 mm	13.1 mm	13.5 mm
11	2.3 mm	2.5 mm	2.0 mm	2.0 mm	12.9 mm	12.5 mm
12	2.2 mm	2.5 mm	1.5 mm	2.4 mm	12.7 mm	12.8 mm
Mean ± SD	2.15 ± 0.49	2.48 ± 0.33	1.73 ± 0.41	2.42 ± 0.34	13.18 ± 1.22	13.65 ± 1.25

Differences in measurement at 6 and 8 mm for buccal cortical plate thickness (P < 0.05) and buccolingual bone thickness (P < 0.05) were found to be significant, whereas for mesiodistal width it was insignificant (P > 0.05) (Table 2). The error of the method (EM) for mesiodistal bone width, buccal and palatal cortical plate thickness and buccopalatal bone depth measurements were found to be 0.40, 0.58 and 0.48, respectively (Table 3).

**Table 2 -:** Paired t-test for mesiodistal bone width, buccal and palatal cortical plate thickness and buccolingual bone depth measurements.

	Mesiodistal width	Buccal cortical plate thickness	Buccolingual depth
T-test value	1.76	3.37	2.51
P value	0.13 (N.S., P > 0.05)	0.021 (Sig, P < 0.05)	0.044 (Sig, P < 0.05)

**Table 3 -:** Error of the method for mesiodistal bone width, buccal and palatal cortical plate thickness and buccolingual bone depth measurements at 6 and 8 mm.

	Mesiodistal width	Buccal cortical plate thickness	Buccolingual depth
Error of the method	0.40	0.58	0.48

## DISCUSSION

Miniscrews^13^^-^ 17 are now frequently used for establishing absolute anchorage for orthodontic tooth movement. They are easily inserted and removed without a mucoperiosteal flap, and can be loaded immediately after insertion.^18^ Their potential applications include improving anchorage, increasing the horizontal component of force applied during space closure, posterior intrusion in open-bite cases, distalization of molars, extrusion of impacted teeth, molar uprighting and correction of midline diastema.^7^^,^ 8 ^,^ 18

The mandibular buccal region had the thickest cortical bone of all evaluated regions. Thicker cortical bone has been previously reported in the mandible than in the maxilla.^12^^,^ 19 Increased cortical bone thickness and higher bone mineral density have been shown in the mandibular buccal region when compared to the maxillary buccal and lingual regions,^20^^-^ 23 as the mandible is found to be always under torsional and bending strains or forces, whereas the maxilla is generally subjected to more compressive forces.^24^ Also, in animal experiments, it has been demonstrated that regions which experience higher strain during function develop thicker cortical bones.^25^

Thus, in humans, cortical bone in the mandibular buccal region was found to be thicker posteriorly, and it becomes progressively thinner anteriorly.^12^^,^ 26 This pattern might also be explained by the higher functional demands placed on posterior teeth. Van Eijden^24^ reported an increase in the longitudinal elastic modulus (increase in stress per unit of strain) between the molar region and the symphysis. Stress and strain differences could give rise to the differences in cortical thickness in this region.

Age-related differences between younger, adolescent and older patients in cortical bone thickness might be explained by changes in functional capacity, because maximum bite forces, masticatory muscle size, and muscle activity have the tendency to increase with age. Changes in the functional capacity, which alter biomechanical stresses and strains, have shown to manipulate cortical bone thickness and bone density because increased strains and stresses within a certain limit increase cortical bone thickness and bone mineral density.^10^

In the mandible, the safest sites for miniscrew insertion have been found to be between the first and second molars, first and second premolars, first molar and first premolar and first premolar and canine. These sites provide moderate inter-radicular space and sufficient cortical plate thickness. However, due to root proximity, the area suitable for miniscrew insertion is over 8 mm from the alveolar crest.^6^

In this study, the CEJ was selected as the starting point for measurements, as compared to other studies in which alveolar crest was used, which could be affected by different periodontal problems.^27^^,^ 28 The maximum level of measurement in this study was selected to be 6 and 8 mm from CEJ because miniscrew placement is most commonly advised in the area of attached gingiva.^29^

The selection of proper miniscrew diameter and length is important as it may hamper eruption or deflect erupting premolars during mixed dentition. Hence, selection will depend upon inter-radicular mesiodistal bone width, buccal cortical plate thickness and bucco-lingual bone depth.^28^ Currently, most miniscrews have diameters ranging from 1.2 to 2 mm. Presently, there are no relative data available on the amount of bone that is to be present between miniscrews and dental roots for both periodontal health and miniscrew stability. Considering that the width of the periodontal ligament is approximately 0.25 mm, it is assumed that a minimum clearance of 1 mm of alveolar bone around the screw could be sufficient for periodontal health.^6^^,^ 28 Combining this value with our data, the safe zone for a miniscrew 1.2 mm in diameter, placed in the inter-radicular spaces have been identified to be at 8 mm.

Radiographic analysis is a pre-requirement in determining anatomic sites for implant placement. Three-dimensional imaging techniques, such as CT or MRI imaging, have turned into important diagnostic imaging in the head and neck.^30^ CT involves a considerably higher radiation dose^31^ in comparison to conventional radiography, as well as high working costs and considerable investment in equipment.^32^ Digital volume tomograph (DVT) is a new imaging technique which produces three-dimensional images similar to CT, but at a low radiation dose which is comparable with panoramic radiograph, and at a lesser cost. DVT technology in clinical practice has numerous advantages, such as image accuracy, rapid scan time and display modes which are unique to maxillofacial imaging. Three-dimensional volumetric tomograph is also well suited for imaging the craniofacial area because it provides clear images of highly contrasted structures which are extremely useful for evaluating bone.^33^^,^ 34 Hence, in this study, three-dimensional digital volumetric tomograph (DVT) was used to assess mesiodistal bone width, cortical bone thickness and buccolingual bone depth.

In the mandibular molar region, mini-implants placement between premolars is not recommended due to the presence of mental foramen.^29^ Hence, the proximity of the mental foramina and bone density in the posterior region needs to be assessed in mixed dentition in order to provide a three-dimensional analysis for miniscrew placement. However, the results of the present study need to be correlated with clinical assessment so as to maintain optimum periodontal health and miniscrew stability.

## CONCLUSION

After evaluating the amount of bone thickness in the inter-radicular spaces of the mandibular posterior region, the results of the present study show promising evidence for safe miniscrews placement in the mixed dentition period. This results need to be reevaluated in a larger scale.

Miniscrew has proved to be the best alternative to routinely use clinical appliances for space management, uprighting and distalization of molars, and intrusion and extrusion of teeth. It can also be used as a temporary prosthesis abutment in younger patients.
